# FOGS: A SNPSTR Marker Database to Combat Wildlife Trafficking and a Cell Culture Bank for *Ex‐Situ* Conservation

**DOI:** 10.1111/1755-0998.14062

**Published:** 2025-01-10

**Authors:** Annika Mozer, Camilla Bruno Di‐Nizo, Albia Consul, Bruno Huettel, Richard Jäger, Ayodélé Akintayo, Christoph Erhardt, Lena Fenner, Dominik Fischer, Sophia Forat, France Gimnich, Peter Grobe, Sebastian Martin, Vikram Nathan, Ammar Saeed, Laura von der Mark, Christian Woehle, Klaus Olek, Bernhard Misof, Jonas J. Astrin

**Affiliations:** ^1^ Leibniz Institute for the Analysis of Biodiversity Change Museum Koenig Bonn Germany; ^2^ Max‐Planck‐Genome‐centre Cologne Max Planck Institute for Plant Breeding Research Köln Germany; ^3^ Department of Natural Sciences, Institute for Functional Gene Analytics Bonn‐Rhein‐Sieg University of Applied Sciences Rheinbach Germany; ^4^ Zoo Wuppertal Wuppertal Germany; ^5^ Labor für Forensische Analytik Leverkusen Germany; ^6^ Faculty of Science, McGill University Montreal Quebec Canada

**Keywords:** biobanking, cryopreservation, database, illegal wildlife trade, SNPSTR, wildlife forensics

## Abstract

Illegal wildlife trade is a growing problem internationally. Poaching of animals not only leads to the extinction of populations and species but also has serious consequences for ecosystems and economies. This study introduces a molecular marker system that authorities can use to detect and substantiate wildlife trafficking. SNPSTR markers combine short tandem repeats with single nucleotide polymorphisms within an amplicon to increase discriminatory power. Within the FOGS (Forensic Genetics for Species Protection) project, we have established SNPSTR marker sets for 74 vertebrate species. On average, each set consists of 19 SNPSTR markers with 82 SNPs per set. More than 1300 SNPSTR markers and over 300 STR markers were identified. Also, through its biobanking pipeline, the FOGS project enabled the cryopreservation of somatic cells from 91 vertebrate species as well as viable tissues for later cell initiation from a further 109 species, providing future strategies for *ex situ* conservation. In addition, many more fixed tissues and DNA samples of endangered species were biobanked. Therefore, FOGS was an interdisciplinary study, combining molecular wildlife forensics and conservation tools. The SNPSTR sets and cell culture information are accessible through the FOGS database (https://fogs‐portal.de/data) that is open to scientists, researchers, breeders and authorities worldwide to protect wildlife from illegal trade.

**Abbreviations**
BOLD: barcode of life data system
bp: basepair
CBD: convention on biological diversity
CITES: Convention on International Trade in endangered species of wild fauna and flora
COI: cytochrome C oxidase subunit 1
DMSO: dimethyl sulfoxide
DNA: deoxyribonucleic acid
FBS: fetal bovine serum
FOGS: forensic genetics for species protection
HBSS: Hank's balanced salt solution
IUCN: International Union for Conservation of Nature
IWT: Illegal Wildlife Trade
LIB: Leibniz Institute for the Analysis of Biodiversity Change
NCBI: National Center for Biotechnology Information Database
PCR: polymerase chain reaction
SNP: single nucleotide polymorphism
STR: short tandem repeats
UNODC: United Nations on Drugs and Crimes

## 1 Introduction

The World Bank estimates that illegal logging, fishing and wildlife trade account for at least one trillion USD per year (World Bank 2019). This makes illegal wildlife trade (IWT) one of the most lucrative illicit markets worldwide (van Uhm 2016; Wyatt 2014). The consequences are severe and far‐reaching including impacts on global biodiversity (Fukushima, Mammola, and Cardoso 2020) and a heightened risk of introducing invasive species (García‐Díaz et al. 2017), animal diseases (Costard et al. 2013) or zoonoses (Chomel, Belotto, and Meslin 2007), human injury and fatality (Prakash et al. 2021) or threats to national security (Wyatt 2013).

The United Nations Office on Drugs and Crime recorded that nearly 6000 different species have been confiscated since the inception of the World Wildlife Seizures (World WISE) database (UNODC 2020). However, as these data are based on seizures, it is likely that many species are under‐reported or even undetected (Symes et al. 2018). Nevertheless, no more than 5% of all seizures can be attributed to a single species (UNODC 2020). This highlights that IWT is a species‐wide problem and not limited to only a few charismatic species (Phelps, Biggs, and Webb 2016). Therefore, tools to detect, investigate, prosecute and prevent IWT are also needed for a wide range of species.

As short tandem repeats (STRs or microsatellites) are the prevailing and already validated DNA markers for identification purposes in human forensics (EU 2009; Hares 2015) they have also been used in some legal cases of IWT (e.g., Rodionov et al. 2021; White et al. 2012). However, the development of suitable STR marker sets requires a considerable amount of time and resources and as such, there are not many marker sets available in relation to the number of species that require such tools now (Alacs et al. 2010; Johnson, Wilson‐Wilde, and Linacre 2014). Single nucleotide polymorphisms (SNPs), in contrast, are more common in the academic world and have been used for niche applications in human and wildlife forensics (Butler Gettings et al. 2014; Ogden and Linacre 2015). Although SNPs are mostly biallelic (Phillips et al. 2020) and thus provide less information per marker, they are much more abundant in the genome. For example, 84.7 million SNPs have been identified in the human genome, compared to only 60,000 structural variants including STRs (The 1000 Genomes Project Consortium 2015).

SNPSTR markers combine the properties of STRs and SNPs, as each marker consists of an STR and at least one SNP within the flanking regions (~400 basepair [bp] up‐ or downstream) of the STR, such that the combined marker can be amplified by one PCR (Mountain et al. 2002). Due to the physical proximity of the STR and SNPs, complete linkage between them can be assumed (Ramakrishnan and Mountain 2004). By combining the information from both types of genetic variation within one marker, it is possible to resolve STR isoalleles of the same length (Mountain et al. 2002; Mozer et al. 2024; Ramakrishnan and Mountain 2004) as well as to increase the discriminatory power and therefore more precise forensic statistical values are achieved (Mozer et al. 2024). Due to the different mutation rates of SNPs and STRs, the combined SNPSTRs are better suited for lineage reconstructions (Mozer et al. 2024). The differentiation of unbalanced alleles and PCR artefacts is also improved due to the usage of flanking SNPs (Mozer et al. 2024). To achieve sufficiently high statistical power, a set of several SNPSTRs needs to be analysed (Mountain et al. 2002; Mozer et al. 2024). Such multiplex analysis of SNPSTRs is best achieved via high‐throughput sequencing (HTS; also known as next generation sequencing, NGS, or massively parallel sequencing, MPS), as multiple flanking SNPs cannot be revealed by length‐based systems such as capillary electrophoresis. High‐throughput sequencing has the further advantage that, unlike capillary electrophoresis, no allele standards need to be established for a standardised allele identification, as sequenced SNPSTRs only need a reference sequence. In addition, SNPSTRs are highly versatile, as they can be used for both STR and SNP applications without the need to develop and validate a new set for each variant type.

Biobanks, institutions that guarantee the integrity, authenticity, availability and (where necessary) confidentiality of molecular and/or viable samples and their data (Astrin, Zhou, and Misof 2013), have been used as a key resource to access and to archive voucher samples and their importance to wildlife conservation and IWT has been increasingly recognised (Colella et al. 2020; Pérez‐Espona 2021). Against the backdrop of the global biodiversity crisis along with high rates of illegal animal trafficking, biodiversity biobanks are intensifying their efforts to cryopreserve cell cultures. Viable cells are an expandable resource as they can be repeatedly thawed, regrown and stored again, and cell culture is an essential tool in almost all fields of biology (Corrales and Astrin 2023; Ryder and Onuma 2018). Cell culture provides in principle unlimited access to high‐quality DNA, RNA or proteins as well as to chromosomes, interphase nuclei and other biomolecules which can then be used in a wide range of genomic and evolutionary studies and help to characterise biological diversity (Ezaz et al. 2008). Somatic cells also constitute a potential genetic resource for the conservation of species and maintenance of biodiversity (Leon‐Quinto et al. 2009; Bolton et al. 2022; Mooney et al. 2023; Praxedes et al. 2018), as they can be reprogrammed into induced pluripotent stem cells (Ben‐Nun et al. 2011). Cryobanking of viable cell material, especially those identified as threatened with extinction in the near future, is essential for future *ex situ* conservation strategies (Mooney et al. 2023).

The project Forensic Genetics for Species protection (FOGS) started in 2020 and was concluded in 2024 and had the aim to (i) develop SNPSTR marker sets for several species, (ii) to cryopreserve cell cultures and archive fixed biomaterial of species relevant to IWT, and (iii) to establish an openly accessible SNPSTR marker and cells database. Forensic scientists and researchers, customs, law enforcement and breeders can access the database via the FOGS portal (https://fogs‐portal.de/en) and adapt the markers to their purposes (e.g., for use in combating IWT, to validate the legitimacy of breeds or to characterise biodiversity). Here we present the FOGS project and inform on the SNPSTR marker sets developed, the species for which cells and tissues have already been cryopreserved and the FOGS database as a bioinformatics hub. Although FOGS was finalised, this project provided the establishment of the cell culture bank at the Leibniz Institute for the Analysis of Biodiversity Change (LIB), Museum Koenig, which will be continually expanded.

## 2 Material and Methods

### 2.1 Samples

Species were selected based on consultation with nature conservation and/or law enforcement authorities, availability, relevance to IWT and conservation status. Requirements imposed by the Convention on International Trade in Endangered Species of Wild Fauna and Flora (CITES 2023b) and the Nagoya Protocol on Access to Genetic Resources and the Fair and Equitable Sharing of Benefits Arising from their Utilisation to the Convention on Biological Diversity (CBD 2015) have been met. A list of sample donors can be found in Table [Link], [Link]. Animals were handled following the ethical guidelines of the collaborators' institutions.

On average, 10 samples per species were used for SNPSTR marker development. Four species sets were developed based on more than 10 samples and 18 species sets were developed based on fewer than 10 samples due to sample availability or quality. For cells, fresh tissues (preferably skin or eye) from different species were obtained from *post‐mortem* individuals (freshly dead) or opportunistically during veterinary procedures (e.g., parts of feathers, parts of tissues that have been removed during indicated veterinary surgery). In this regard, no animal was caught, restrained, manipulated or suffered any kind of pain for the purpose of the study and therefore procedures did not require additional animal welfare permission according to local Animal Welfare Law. In some cases, whole carcasses were prepared as museum voucher specimens and stored at the LIB, Museum Koenig.

DNA was extracted using the BioSprint 96 Blood and Tissue Kit (Qiagen, Hilden, Germany) or the DNeasy Blood and Tissue Kit (Qiagen) and stored in the Biobank of the LIB (contingent on donor approval). The species of each sample used for SNPSTR development and/or cell culture was confirmed by barcoding using the mitochondrial cytochrome c oxidase subunit 1 (COI) gene with primers LCO1490‐JJ (5′‐CHACWAAYCATAAAGATATYGG‐3′) and HCO2198‐JJ (5′‐AWACTTCVGGRTGVCCAAARAATCA‐3′; Astrin and Stüben 2008) and the Barcode of Life Data System (BOLD; Ratnasingham and Hebert 2007). Samples were quantified using a Quantus fluorometer (QuantiFlour ONE dsDNA System; Promega, Fitchburg, USA) and if the concentrations determined were less than 10 ng/μL, 100 μL of extracted DNA were concentrated on a Savant SPD111V vacuum concentrator (Thermo Scientific, Waltham, USA) at 35°C for 30 min.

### 2.2 Whole Genome Sequencing and STR Filtering

If a reference genome was available on NCBI, this assembly was used (*n* = 27, see Table S2 for GenBank accession numbers). Otherwise, samples were sequenced on a Sequel II (PacBio, Menlo Park, USA; Max Planck Genome‐Centre, Cologne, Germany) following the ultra‐low input protocol (Schneider et al. 2021; *n* = 25) or on an Illumina MiSeq (Reagent Kit v3 (600‐cycle); San Diego, USA; Macrogen, Amsterdam, the Netherlands and MP‐GC; *n* = 22). Both methods included a fragmentation step before library preparation. These sequenced genomes were assembled using SPAdes, with paired end reads used for the Illumina‐derived sequences (v. 3.15.5; Prjibelski et al. 2020). These sequencing steps were aimed at identifying suitable repetitive sequences, which were later confirmed by amplicon sequencing and thus established as markers (see Mozer et al. 2024). As described in Mozer et al. (2024) PERF (v. 0.4.6; Avvaru, Sowpati, and Mishra 2018), BEDTools (v. 2.27.1; Quinlan and Hall 2010) and CD‐HIT (v. 4.8.1; Fu et al. 2012) were used to extract sequences containing tetranucleotide STRs with 11–20 repeats but no other repetitive sequence within a 170 bp flanking region up‐ and downstream of the STR.

### 2.3 PCR, Library Preparation and Amplicon Sequencing

Following Mozer et al. (2024), up to 30 primer‐pairs for one multiplex reaction per species were designed using PrimerPlex (v. 2.76, Premier Biosoft, San Francisco, USA) and synthesised by Metabion (Planegg, Germany). Temperature gradients (60°C ± 3°C) were then performed using the Multiplex PCR Kit (Qiagen, Hilden, Germany) on a Biometra TGradient Thermocycler (Analytik Jena, Jena, Germany; 95°C 15 min, [95°C 30 s, *T*_A_ 1:30 min, 72°C 1:30 min] for 35 cycles, 72°C 10 min, 4°C ∞). Agarose gels (1.5%, 100 V, 60 min) were used to determine optimal annealing temperatures (as judged by the brightest signals) and to assess possible primer dimers for the PCR multiplex (BioDocAnalyze, Biometra). PCRs (same conditions as described above) were then performed on all samples obtained per species. The amplicons were processed on an automated liquid handling G3 workstation (PerkinElmer, Waltham, USA) with NEBNext Ultra II DNA Library Prep Kit for Illumina (New England Biolabs, Ipswich, USA) to prepare Illumina‐compatible libraries. Libraries were fluorometrically quantitated (Quantus fluorometer, Promega) subsequently, equimolar pooled and sequenced on a MiSeq sequencer (Reagent Kit v3 (600‐cycle); Illumina) at the Max Planck Genome‐Centre Cologne.

### 2.4 Analysis

Sequences were trimmed using fastp (v. 0.20.0, minimum length 100 bp and phred quality of ≥ Q15; Chen et al. 2018), mapped using bwa mem (v. 0.7.17; Li 2013) and processed using samtools (v. 1.15.1; Danecek et al. 2021). STRs were analysed using STRaitRazor (v. 3.01; Woerner, King, and Budowle 2017), while SNPs were identified using standard hard filtering and following other GATK best practice recommendations (v. 4.2.6.1; McKenna et al. 2010; van der Auwera and O'Connor 2020). A SNPSTR marker was selected if the STR showed variability (i.e., at least two different STR alleles were called) and at least one SNP was identified within the flanking region (a SNP within the STR was considered as STR variability and not as a flanking SNP). The heterozygosity of the STRs was not taken into account when selecting SNPSTRs, as flanking SNPs could also resolve isoallelic STRs. The SNPSTR alleles had a read depth of ≥ 10. Primer and SNPSTR data can be found in Tables S3 and S4, respectively, while sequencing data are also available from NCBI (Bioproject ID: PRJNA954578).

### 2.5 Cell Culture

Tissue samples obtained from very freshly deceased animals, or blood feathers, were washed twice in Hank's balanced salt solution (HBSS, Gibco, Waltham, USA) supplemented with antibiotic (100 U/mL penicillin and 100 g/mL streptomycin; Sigma‐Aldrich, St. Louis, USA) and antimycotic (2.5 μg/mL amphotericin B; Biowest, Nuaillé, France), transferred to a Petri dish and minced with sterile scissors and scalpels. The tissues were then used to obtain primary cells. Alternatively, tissues were frozen gradually (1°C per minute) and stored for later culture initiation in liquid nitrogen vapour phase below −190°C in freezing medium: 10% fetal bovine serum (FBS, Biowest) and 10% dimethyl sulfoxide (DMSO; Sigma‐Aldrich) in plain base media (Houck, Lear, and Charter 2017; Wong et al. 2012).

Primary cells were obtained from small tissue fragments or from free cells after enzymatic digestion with collagenase (0.125 mg/mL, Roche, Basel, Switzerland), according to standard protocols with modifications (Freshney 2010; Houck, Lear, and Charter 2017; Masters 2000). Flasks were incubated in a controlled environment at appropriate temperatures and cell media, depending on the taxonomic group (see Table S5). After reaching confluence (monolayer covering approximately 80% of the flask), cells were subcultured with 0.125% trypsin solution (Biowest) and subsequently gradually frozen (1°C per minute) at −190°C using cryoprotectant medium DMSO (Sigma‐Aldrich) 10% plus 90% base medium (see Table S5) supplemented with 10%–20% FBS (Biowest).

### 2.6 Database

The FOGS data portal is written in Python 3.10 and uses the Pyramid web framework (https://trypyramid.com/). The portal application itself runs in a Docker container in the Docker Swarm environment of the LIB (https://fogs‐portal.de/data/). The data infrastructure was built on the existing specimen occurrence data management structure at the LIB (Grobe et al. 2019; https://datacenter.leibniz‐lib.de/wiki/dataflow:general_dataflow) and covers the complete data lifecycle from acquisition to archiving and publication of the data, (e.g., in GBIF; https://gbif.org or the biodiversity biobanks network GGBN; Droege et al. 2016). The data infrastructure consists of four programs and tools that read data from standardised tables, containing all SNPSTR information provided by the laboratory, into the DiversityCollection (part of the DiversityWorkbench suite of collection databases used at the LIB) and merge them with existing information on the occurrence and identification of the specimen. A data integration program reads the data and makes it available on the data portal. All programs developed are available in the LIB's Gitlab repositories: https://gitlab.leibniz‐lib.de/FOGS/. For more information, please see also Table S6.

## 3 Results

### 3.1 Establishment of SNPSTR Markers for 74 Species

We established SNPSTR marker sets for 74 species: 45 birds, 17 reptiles, five fishes, four mammals and three amphibian species (Table 1). Of these, 32 species are covered by CITES Appendices (3 I, 21 II, 9 III; CITES 2023a). A total of 1365 SNPSTR markers were identified, with an average of 19.23 ± 5.62 markers per species, all of which can be amplified in a single multiplex PCR reaction per species. On average, each SNPSTR amplicon harboured 4.13 ± 2.27 SNPs and 82.30 ± 53.51 SNPs were contained in each marker set. On average, 10.11 ± 3.79 alleles were found per SNPSTR marker and an average heterozygosity rate of 0.69 ± 0.26. Because variable STRs without a flanking SNP already contain useful information and can be used for forensic and research purposes, 314 STR‐only markers were also included (5.08 ± 1.82 length‐based alleles per STR loci with a heterozygosity of 0.56 ± 0.30). Information on all identified loci (primer sequences, number of alleles) is provided by the FOGS database (https://fogs‐portal.de/data/) and can be found in the Tables S3 and S4 of this manuscript.

**TABLE 1:** List of species and taxa covered in the FOGS project. For each species, the number of SNPSTR markers is given as well as the cell culture with either viable somatic cells or tissue for later cell initiation. Species are sorted alphabetically by taxa.

	SNPSTR marker	STR marker	Cell culture
**Amphibians**			
*Blommersia transmarina*			Viable tissue
*Bombina bombina*			Viable tissue
*Bombina variegata*			Viable tissue
*Calotriton asper*	5	2	Viable tissue
*Discoglossus scovazzi*			Viable tissue
*Hyla arborea*	3	2	
*Ichthyosaura alpestris*			Cells
*Polypedates otilophus*			Viable tissue
*Salamandra atra*	12	3	
*Staurois guttatus*			Viable tissue
*Theloderma albopunctatum*			Viable tissue
*Theloderma corticale*			Viable tissue
*Zhangixalus dennysi*			Viable tissue
**Birds**			
*Acanthis flammea*	23	1	
*Accipiter gentilis*	14	13	
*Accipiter nisus*	21	4	
*Acrocephalus scirpaceus*	21	1	
*Aegolius funereus*			Cells
*Alauda arvensis*	21		
*Alcedo atthis*	12	15	
*Alopochen aegyptiaca*			Cells
*Aptenodytes patagonicus*			Viable tissue
*Anodorhynchus hyacinthinus*			Cells
*Anodorhynchus leari*			Cells
*Aquila chrysaetos*	16	2	Cells
*Aquila nipalensis*			Cells
*Aratinga solstitialis*			Viable tissue
*Athene noctua*	20	8	Viable tissue
*Bombycilla garrulus*	20	5	
*Branta sandvicensis*			Viable tissue
*Bubo bubo*	13	17	Cells
*Bubo scandiacus*			Viable tissue
*Bubo virginianus*			Viable tissue
*Burhinus capensis*			Viable tissue
*Buteo auguralis*			Viable tissue
*Buteo buteo*			Cells
*Cacatua galerita*			Cells
*Cacatua sulphurea*			Cells
*Carduelis carduelis*	21	1	
*Cariama cristata*			Viable tissue
*Cathartes aura*			Viable tissue
*Chiroxiphia caudata*			Cells
*Chloris chloris*	23	3	
*Ciconia nigra*			Cells
*Cinclus cinclus*	19	5	
*Circus aeruginosus*			Cells
*Coccothraustes coccothraustes*	23	4	Viable tissue
*Copsychus malabaricus*			Viable tissue
*Cyanerpes caeruleus*			Viable tissue
*Cyanerpes cyaneus*			Viable tissue
*Cyanoramphus novaezelandiae*			Viable tissue
*Dacelo novaeguineae*			Viable tissue
*Dendrocopos major*	20	5	Cells
*Dryocopus martius*	12	11	
*Emberiza citrinella*	26	1	
*Entomyzon cyanotis*			Viable tissue
*Erithacus rubecula*			Viable tissue
*Erythrura gouldiae*			Viable tissue
*Eudocimus ruber*			Viable tissue
*Euphonia violacea*			Viable tissue
*Falco peregrinus*			Cells
*Falco subbuteo*			Cells
*Falco tinnunculus*			Cells
*Ficedula hypoleuca*	24	1	
*Fringilla coelebs*	24		Viable tissue
*Fringilla montifringilla*	19		Cells
*Furnarius leucopus*			Viable tissue
*Grus grus*	25	3	
*Gallicolumba luzonica*			Cells
*Gallus gallus domesticus*			Cells
*Geranoaetus polyosoma*			Viable tissue
*Geronticus eremita*			Cells
*Haematopus ostralegus*	12	7	
*Haliaeetus leucocephalus*			Cells
*Hirundo rustica*			Viable tissue
*Lanius collurio*	26	1	
*Leucopsar rothschildi*			Viable tissue
*Limosa limosa*			Cells
*Linaria cannabina*	22	1	
*Liocichla ameiensis*			Viable tissue
*Milvus migrans*			Cells
*Milvus milvus*			Viable tissue
*Motacilla alba*	23		
*Muscicapa striata*	28		
*Myiopsitta monachus*			Cells
*Nestor notabilis*			Cells
*Numenius arquata*	22	5	
*Nymphicus hollandicus*			Viable tissue
*Oenanthe oenanthe*	23	2	
*Oriolus oriolus*			Viable tissue
*Parabuteo unicinctus*			Cells
*Parus major*			Viable tissue
*Passer domesticus*			Viable tissue
*Pelecanus onocrotalus*			Viable tissue
*Phasianus colchicus*			Cells
*Philemon citreogularis*			Viable tissue
*Phoenicopterus chilensis*			Viable tissue
*Phoenicopterus ruber*			Viable tissue
*Phylloscopus collybita*	25	2	
*Phylloscopus trochilus*	24	1	
*Picus viridis*	11	14	Cells
*Pitangus sulphuratus*			Viable tissue
*Pitta sordida*			Viable tissue
*Platycercus elegans*			Viable tissue
*Ploceus jacksoni*			Viable tissue
*Prunella modularis*			Viable tissue
*Psittacula krameri*	23	6	Cells
*Psittacus erithacus*	14	14	
*Pseudastur albicollis*			Cells
*Ptilinopus pulchellus*			Cells
*Pycnonotus xanthopygos*			Viable tissue
*Pygoscelis papua*			Viable tissue
*Pyrrhula pyrrhula*	20	2	
*Ramphocelus bresilius*			Viable tissue
*Recurvirostra avosetta*	16	4	Viable tissue
*Regulus ignicapilla*			Viable tissue
*Rhea americana*			Viable tissue
*Scolopax rusticola*			Cells
*Serinus serinus*	28		
*Sitta europaea*	22		Viable tissue
*Somateria spectabilis*			Viable tissue
*Spheniscus demersus*			Cells
*Spinus spinus*	20	2	
*Stephanoaetus coronatus*			Cells
*Strix aluco*			Viable tissue
*Strix uralensis*			Cells
*Sturnus vulgaris*			Cells
*Sylvia atricapilla*	25	2	
*Taeniopygia guttata*			Viable tissue
*Tangara gyrola*			Viable tissue
*Tangara icterocephala*			Viable tissue
*Tangara mexicana*			Cells
*Terathopius ecaudatus*			Cells
*Tringa totanus*	23	3	
*Troglodytes troglodytes*			Viable tissue
*Turdus iliacus*	19	4	
*Turdus merula*			Cells
*Turdus philomelos*	20	4	Cells
*Turdus viscivorus*	9	1	
*Tyto alba*			Cells
*Vanellus vanellus*	22	4	
*Zamenis situla*	14	11	
*Zenaida graysoni*			Cells
**Fish** ^a^			
*Abramis brama*			Cells
*Acipenser gueldenstaedtii*	25		
*Acipenser stellatus*	20	6	
*Anguilla anguilla*	20		Cells
*Anguilla rostrata*	23		
*Cyprichromis coloratus*			Viable tissue
*Cyprichromis leptosoma*			Viable tissue
*Cyprichromis microlepidotus*			Viable tissue
*Gnathochromis pfefferi*			Viable tissue
*Huso huso*	16	6	
*Oryzias eversi*			Cells
*Paracyprichromis nigripinnis*			Viable tissue
*Phoxinus phoxinus*			Cells
*Simochromis diagramma*			Cells
*Valencia hispanica*			Viable tissue
*Vimba vimba*			Viable tissue
**Mammals**			
*Acinonyx jubatus*			Viable tissue
*Ammotragus lervia*			Cells
*Apodemus sylvaticus*			Viable tissue
*Bison bonasus*			Viable tissue
*Bos taurus*			Cells
*Budorcas taxicolor*			Cells
*Capra sibirica*			Viable tissue
*Capreolus capreolus*			Viable tissue
*Canis aureus*			Cells
*Canis lupus*			Cells
*Catopuma temminckii*			Viable tissue
*Cebuella pygmaea*	15	6	
*Cervus elaphus*			Viable tissue
*Choloepus didactylus*			Cells
*Choloepus hoffmanni*			Cells
*Cricetus cricetus*	9	16	
*Crocidura russula*			Cells
*Cuon alpinus*			Cells
*Diceros bicornis*			Viable tissue
*Dicotyles tajacu*			Cells
*Dolichotis patagonum*			Viable tissue
*Equus caballus przewalskii*			Cells
*Erinaceus concolor*			Cells
*Felis silvestris*			Cells
*Gazella dorcas*			Cells
*Gorilla gorilla*			Cells
*Helarctos malayanus*			Cells
*Herpailurus yagouaroundi*			Cells
*Hylobates syndactylus*			Viable tissue
*Inia geoffrensis*			Viable tissue
*Lepus europaeus*			Cells
*Lepus timidus*			Cells
*Loxodonta africana*	11	10	Viable tissue
*Macropus giganteus*			Cells
*Macropus rufogriseus*			Viable tissue
*Madoqua kirkii*			Cells
*Martes foina*			Viable tissue
*Mandrillus leucophaeus*			Viable tissue
*Muntiacus muntjak vaginalis*			Cells
*Myotis blythii*			Cells
*Nasua nasua*			Viable tissue
*Neofelis nebulosa*			Viable tissue
*Okapia johnstoni*			Cells
*Oreamnos americanus*			Cells
*Oryx dammah*			Cells
*Oryx gazella*			Viable tissue
*Ovibos moschatus*			Viable tissue
*Ovis aries*			Viable tissue
*Otocyon megalotis*			Cells
*Pan paniscus*			Cells
*Panthera leo*			Cells
*Panthera tigris*			Cells
*Panthera pardus*			Cells
*Panthera uncia*			Viable tissue
*Phascolarctos cinereus*			Cells
*Phataginus tricuspis*	8	1	
*Pipistrellus pipistrellus*			Viable tissue
*Potamochoerus porcus*			Viable tissue
*Rangifer tarandus*			Viable tissue
*Rhinoceros unicornis*			Cells
*Rhinolophus ferrumequinum*			Cells
*Saimiri sciureus*			Cells
*Suricata suricatta*			Viable tissue
*Sus scrofa*			Viable tissue
*Tamandua tetradactyla*			Viable tissue
*Tragelaphus eurycerus*			Cells
*Tremarctos ornatus*			Cells
*Trichetus manatus*			Cells
*Zalophus californianus*			Viable tissue
**Reptiles** ^a^			
*Acanthosaura capra*			Viable tissue
*Brachylophus vitiensis*			Cells
*Chersina angulata*	20	1	
*Eretmochelys imbricata*	14		
*Gastropholis standingi*			Viable tissue
*Gonocephalus sp*.			Viable tissue
*Lacerta bilineata*	17	5	
*Lacerta viridis*	22	1	
*Laudakia stellio*			Viable tissue
*Malayopython reticulatus*	20	5	
*Morelia viridis*			Cells
*Natrix natrix*	17	3	
*Natrix tessellata*	16	6	
*Ouroborus cataphractus*	10	2	
*Phelsuma standingi*			Viable tissue
*Phrynosoma braconnieri*			Cells
*Phrynosoma taurus*			Cells
*Podarcis muralis*	15	3	
*Python regius*	10	14	
*Sanzinia madagascariensis*			Viable tissue
*Shinisaurus crocodilurus*			Viable tissue
*Thamnophis sirtalis*			Viable tissue
*Terrapene mexicana*	22	4	
*Testudo hermanni*	10	4	
*Uromastyx thomasi*			Viable tissue
*Varanus exanthematicus*	23	1	
*Varanus mitchelli*			Viable tissue
*Varanus niloticus*	18	6	
*Varanus salvator*	14	9	
*Vipera ammodytes*	17	4	
*Vipera berus*	22	4	
∑ Species	74 SNPSTR sets		91 cells + 109 viable tissues

^a^ Not a formal taxonomic group.

### 3.2 Cryopreservation of Cells and Tissues From 91 and 109 Species

From the beginning of the project until the submission of this manuscript, viable cells were cryopreserved from 91 species (including five subspecies—for more details check FOGS portal or LIB portal), comprising 43 birds, 38 mammals, five fishes, four reptiles and one amphibian (Table 1). Of these, viable cells were obtained for four critically endangered species ( *A. anguilla* , *C. sulphurea, G. gorilla*, and *B. vitiensis* ) and one species extinct in the wild ( *Z. graysoni* ; IUCN 2024). In addition to the cultured cells, viable tissues from further 109 species were frozen to ensure that cells could be established in the future. The project allowed the establishment of the LIB cell bank and provided close collaboration with sample providers that continued beyond the end of the project in February 2024. Currently, the cell bank is supported by other third‐party projects and thus the list of species from which cells are obtained is not fixed and grows as we receive new samples. For a more up‐to‐date list, consult the LIB portal.

### 3.3 The FOGS Database and Portal

**FIGURE 1:**
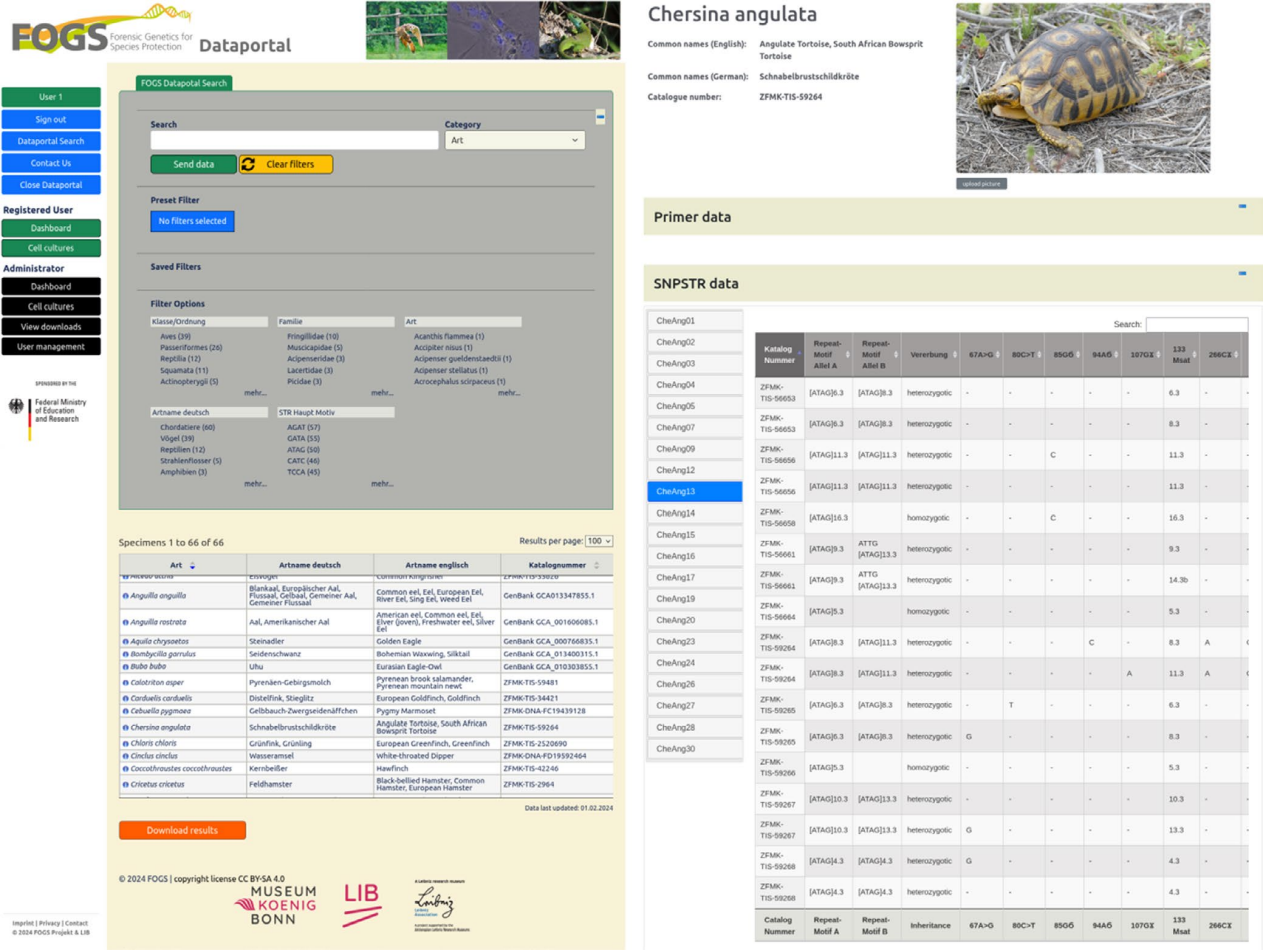
The FOGS data portal (https://fogs‐portal.de/data/). The portal shows SNPSTR marker sets for 74 species, which can be filtered by species name, taxa and STR motifs (left). Details can be shown by clicking on one of the search results (right). The page of *C. angulata* , the beaked turtle, is an example of a detailed view of a species studied in FOGS. The data of the reference individual with the internal catalogue number of the Biobank ZFMK‐TIS‐59265 and the SNPSTR information of the corresponding examined individuals are shown.

**FIGURE 2:**
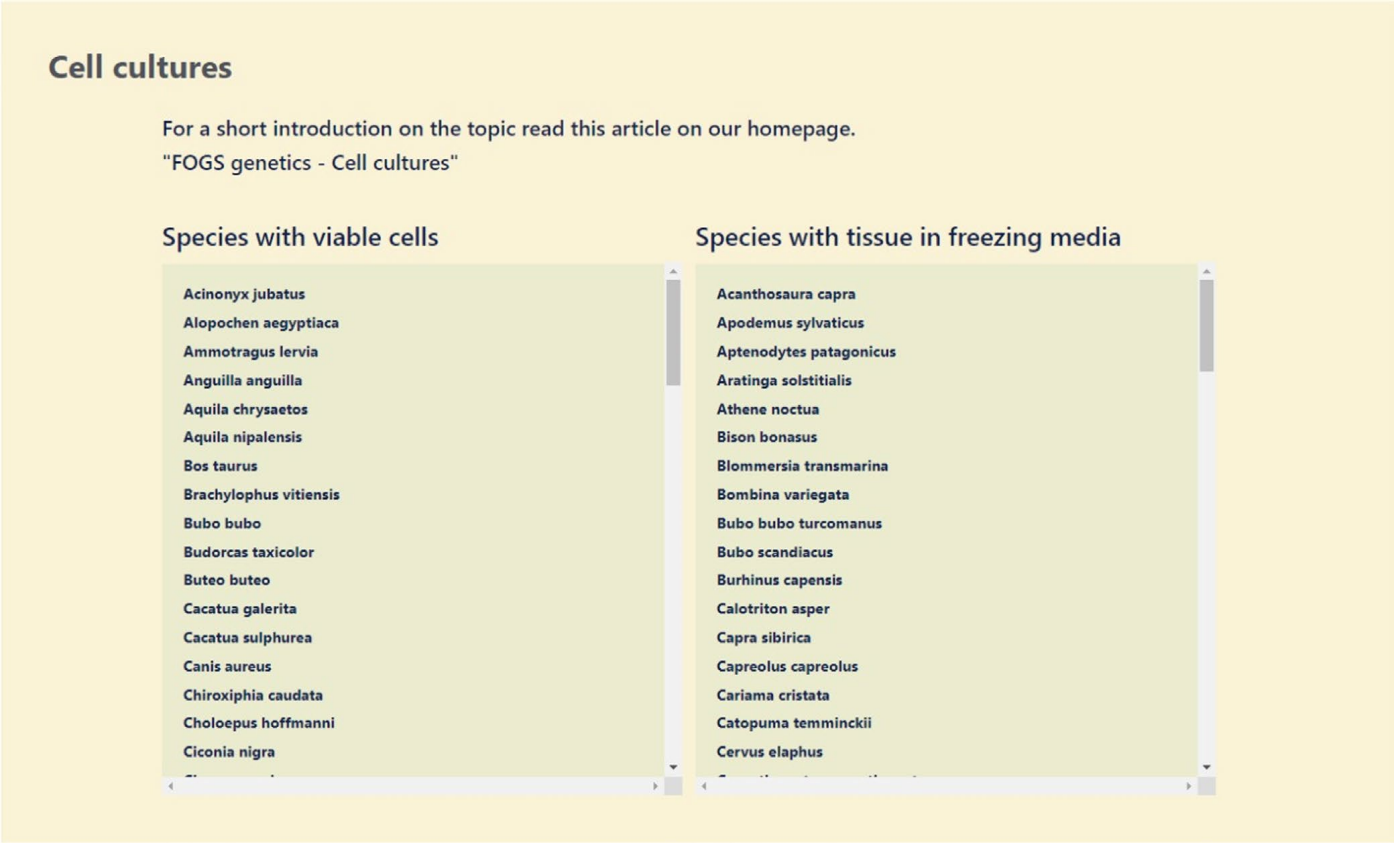
Cell culture list. The portal also shows species with viable cells as well as tissues in freezing media for later cell culturation conserved in the FOGS project.

Based on the data infrastructure elements of the German Barcode of Life project (Geiger et al. 2016), a new data portal (https://fogs‐portal.de/data/) was developed with a focus on easy accessibility to complex data structures for the forensic and research community as well as for authorities. It is accessible free of charge. The data portal has the following features: (1) Search for information on the species being studied, (2) Search for specific categories (species, countries, etc.), and (3) Filter function for the categories: STR repeat motif, country, scientific names, English and German common species names as well as the various taxonomic categories. The result of the search is a list of reference species that matches the search. Clicking on an entry of a reference species in the table opens the detailed view, which displays all information on the species and all associated SNPTRs (Figure 1) as well as cells (Figure 2).

## 4 Discussion

To the best of our knowledge, this project created a first‐of‐its‐kind, interdisciplinary database of SNPSTR markers to combat, detect and substantiate IWT, as well as proper biobanking of samples including setup of cell cultures as an *ex‐situ* conservation strategy for endangered animals. Overall, the FOGS project covers 261 species (Table 1).

The workflow established here can be pursued in a highly efficient and automated fashion. For example, by using bioinformatics primers for a highly multiplexed PCR (up to 30 primer pairs per reaction) can be obtained from any NCBI reference genome within just 30 min. Moreover, we used automated library preparation of a pipetting robot as well as automated bioinformatics analysis and workflows. Furthermore, data can be automatically processed and easily accessed and downloaded using the FOGS data portal. These features of the FOGS project will not only facilitate the implementation and acceptance of the established SNPSTR marker system but are also of particular importance for the time‐critical issue of IWT.

Previous studies assumed that 25%–50% of all human STRs could be revealed as SNPSTRs with further research (Mountain et al. 2002; Ramakrishnan and Mountain 2004). This research indicates that the percentage of SNPSTRs could be significantly higher, considering the fact that we found 1365 SNPSTR markers across different taxa, compared to only 314 STR markers without flanking SNPs and this finding was based on the analysis of just 10 samples per species. Therefore, we hypothesise that SNPSTRs are an abundant marker type and anticipate that SNPSTRs will become more commonly used with the increasing implementation of sequencing technology (Gross, Fleckhaus, and Schneider 2021).

For an effective and legally valid DNA marker, the markers used must be highly informative (e.g., Alacs et al. 2010). On average, we found 19.23 ± 5.62 SNPSTR markers per species (*n* = 74), which seems sufficient compared to other legal wildlife crime cases (e.g., 8 STR loci in Baker et al. 2007, 18 STR loci in Kanthaswamy et al. (2009). Each of these markers contains a variable STR and 4.13 ± 2.27 SNPs. While, by definition, each marker must contain at least one SNP, markers were also found in variable regions (e.g., 19 SNPs within a 325 bp SNPSTR locus; CriCri14). Furthermore, some of the SNPSTR sets developed contain rare cases of tri‐allelic SNPs (e.g., PyrPyr13 35C > G/T or TurPhi21 243C > A/G). As a length‐based analysis of the 1365 SNPSTR markers would only result in 6.13 ± 2.23 alleles, the flanking SNPs and sequencing analysis increased the number of alleles per marker by 3.98 ± 2.82 alleles, a 1.72‐fold increase.

SNPSTRs have proven to be highly useful in parentage testing with parentage exclusion probabilities of over 99.99%, individual identification with respective probabilities of identification extremely exceeding global population sizes, geographic assignment and population assessment (Mozer et al. 2024; Mozer et al. submitted). Further studies may shed light on other applications such as hybrid identification. Species identification via mitochondrial barcodes is the current standard in animals. However, this technique cannot resolve hybrids, as barcodes only identify the maternal line (Linacre et al. 2011). The breeding of hybrids is common practice, e.g., in aviculture (Ottenburghs et al. 2015), but the breeding of several specific hybrid forms have been prohibited in some countries (e.g., hybrids of birds of prey according to the German Federal Species Protection Regulations; Bundesartenschutzverordnung §§8–11). However, as illegal trade in falcons is known to take place (Wyatt 2009, 2011), the ability to identify or to exclude falcon hybrids, for example, would be of great interest for wildlife forensics. Here, future studies using nuclear SNPSTR markers may be able to identify species‐specific SNPs or SNPSTR alleles being private to certain species and thus detect illegal hybrids. Moreover, such SNPSTR alleles might help to detect naturally occurring hybridization of species, which is often suspected but rarely proven in areas where closely related species share habitats. Furthermore, SNPSTRs may be used to confirm species purity prior to translocation and release in species conservation and restoration projects.

A limitation of the current study is the possibility of undetected sequencing errors. As a sequencing error in the reference could lead to the erroneous detection of a SNP, each SNPSTR marker was manually checked before uploading to the FOGS portal. Putative SNPs were removed if the reference sample was re‐sequenced as part of the 10 samples to establish SNPSTR markers and the SNP was not identified in either allele of the reference samples or any of the other samples tested. Moreover, as repetitive structures in DNA can be difficult to sequence (Wenger et al. 2019), the STR component of each SNPSTR amplicon is covered within the maximum forward and reverse sequencing length in the current study. Although we have done our best to account for sequencing errors, we cannot be absolutely certain that all sequencing errors have been identified.

In addition, there is a need for a robust, forensically‐valid bioinformatic tool to reliably detect SNPSTRs in sequencing data. Here, we have conducted the first steps of such an approach with a bioinformatic pipeline. Furthermore, most repetitive structures in the flanking regions of the STRs are filtered out, but if the reference used has only very few repeats, the algorithm sometimes ignores these short repetitive structures, leading to complex STR motifs not being filtered out. This is a particular challenge for automated STR analysis, as most complex STR motifs have to be checked manually.

For use in legal proceedings and other research, the allele frequencies of DNA markers are essential for calculating relevant statistics. However, this was not the scope of the present study. As our database is a collection of SNPSTR markers, it is not an allele frequency database (as opposed to most databases in wildlife forensics, such as Karmacharya et al. 2018; Palsbøll et al. 2006; Wasser et al. 2008). Several studies have shown that establishing allele frequency databases acts as a crime deterrent, leading to a significant reduction in crime rates, while being more cost‐effective than other traditional law enforcement approaches (Anker, Doleac, and Landersø 2021; Doleac 2017). The purpose of our study is to provide experimentally tested markers that can be used, among other things, to establish such allele frequency databases for the species of interest. Therefore, we strongly encourage researchers worldwide to use the SNPSTR markers, as done in Mozer et al. [submitted] and to increase the sample size to optimally address their type of research or forensic question.

Of note, the implementation of SNPSTR markers requires high throughput sequencing technologies that are currently not standard in most wildlife forensic laboratories. However, the SNPSTR markers can also be analysed using conventional fragment length‐based analysis, then not taking advantage of the additional information provided by the flanking SNPs. Moreover, costs for HTS are decreasing with the implementation of nanopore sequencing, an affordable HTS platform with potential applications in wildlife forensic laboratories (Ogden, Vasiljevic, and Prost 2021; Vasiljevic et al. 2021).

Especially in the development of new markers, biobanking is of particular importance. Samples that have been used to establish new markers should be preserved so that in the future, if the markers change, the original samples used to establish previous markers can be re‐analysed to obtain a direct relationship between the old and new markers (as proposed for DNA in Astrin, Zhou, and Misof 2013). Moreover, the need for biobanking in wildlife forensics is highlighted in several publications (Hogg et al. 2018; Pérez‐Espona 2021).

Additionally, both in situ and *ex situ* conservation approaches should be pursued in the light of IWT. The FOGS project provided the perfect opportunity to establish a cell bank at the LIB Biobank, strengthening the small but important global network of viable cell culture repositories focusing on biodiversity (Ryder and Onuma 2018). We have been able to freeze cells and/or tissues of different vertebrate species (some of which are classified as endangered according to the IUCN categories), of species considered to be hosts for different pathogens (e.g., bats) and of species for which basic biological information is still lacking (e.g., almost 35% of the bird species cryopreserved in LIB Biobank lack a formal karyotype description). This achievement was made possible by extensive collaboration with zoos and individual researchers (see Table S1).

Thus, cryopreservation of cells/tissues from a variety of taxonomic groups is essential not only because some studies are species‐specific (e.g., host‐pathogen interactions) but also because it provides a better characterisation of biodiversity and can then be used in a wide variety of genomic and evolutionary studies (Ezaz et al. 2008).

As cells constitute an ample source of high quality DNA and RNA, chromosomes, proteins, etc., establishing primary cell cultures greatly expands the possible range of future sample uses, including future biodiversity conservation initiatives (Mooney et al. 2023; Wong et al. 2012). Currently, new technologies in cell biology (e.g., cloning by somatic cell nuclear transfer and induced pluripotent stem cells) are making the conservation of endangered species increasingly feasible (Ben‐Nun et al. 2011; Loi, Modlinski, and Ptak 2011).

We wish to emphasise the international aspect of IWT, although this study mainly covers European species. In addition, species currently listed as ‘Least Concern’ in the IUCN Red List or not covered by the CITES Appendices have also been included, as some species are also protected by other legislation (e.g., the Commission Regulation 2007; 16 U.S.C. §§ 4901–4916, Wild Bird Population Act 1992) and are therefore still relevant in the fight against IWT. Moreover, even for those species that are currently considered safe, the global trend is clearly one of further deterioration, with overexploitation being one of the major threats to biodiversity (Bellard, Marino, and Courchamp 2022). For example, 69% of wildlife populations are declining (WWF 2022) and the current extinction rate is assumed to be 35 times higher than the expected background extinction rate (Ceballos and Ehrlich 2023).

As other genetic databases (e.g., GenBank [Benson, Lipman, and Ostell 1993], BOLD [Ratnasingham and Hebert 2007], etc), the FOGS Data Portal constitutes a convenient public interface for accessing information on forensically relevant species and can be filtered by specific categories to provide a quick overview of the information available. The single view for an individual species provides detailed information on the reference specimens and species (collecting information, taxonomy, etc.), availability of isolated cells as well as characteristics for forensic analysis using the selected set (primer name and sequence, amplicon sequence and reference allele) and for each SNPSTR locus (STR motif and repeats, SNPs and inheritance status). The data portal is part of a data infrastructure developed within the FOGS project with the aim of managing, archiving and collating all information on the species studied, laboratory results and analyses.

## 5 Conclusion

Through the FOGS project, which ran for 5 years and ended in February 2024, we are now able to provide genetic tools for over 70 species threatened by IWT. The established SNPSTR marker sets can be used for many applications in wildlife forensics and research. The establishment of population databases is still an important step. Nevertheless, as the current study has shown that SNPSTR markers are technically feasible and highly informative, the first step towards population databases, the establishment of marker sets, is made here. The FOGS database is therefore an excellent starting point for laboratories assisting authorities in wildlife crime investigations. As a result, proven IWT can be prosecuted and affected species and populations can be restored. All tissues and/or DNA extracts of the species analysed in FOGS are stored in the LIB Biobank, if not vouchered at another repository. The FOGS project furthermore established a cell bank at the LIB Biobank, enabling the widest possible range of future applications. With the advent of the “Genomic Era” (recently rapid increase in the number of genomes being sequenced including international initiatives aiming to generate genomic resources), cell cultures are ranked as the ideal samples for obtaining top‐quality DNA and RNA and very importantly, allow access to chromosomal information. In addition, they offer opportunities for conservation strategies. Overall, the FOGS project offers two different approaches (in situ and *ex situ*) to protect species and populations from extinction by IWT.

## Author Contributions

J.J.A., K.O., B.M., A.C.: conceptualization. K.O., S.F., A.M.: conceived and designed the experiments. C.B.D.‐N., L.V.D.M., F.G., A.A., J.J.A.: cell culture and biobanking. A.M., L.V.D.M., C.B.D.‐N., A.A., L.F.: performed the experiments. A.M., C.B.D.‐N., A.A.: analysed the data (sequences and cells). A.M., V.N., C.W., S.M.: analysed the data (bioinformatics). P.G., C.E., A.S.: database and portal update. F.G., A.C.: permits. J.J.A., B.M., B.H., R.J., K.O.: contributed reagents/materials/analysis tools/expert knowledge. A.C.: coordination and financial. A.C., J.J.A., D.F., C.B.D.‐N., L.V.D.M.: sample acquisition. J.J.A.: supervision. A.M., C.B.D.‐N.: wrote original draft. All: reviewed the manuscript.

## Conflicts of Interest

The authors declare no conflicts of interest.

## Benefit‐Sharing Statement

The requirements of CITES and the Nagoya Protocol on Access to Genetic Resources and Fair and Equitable Sharing of Benefits Arising from their Utilisation under the CBD were met. Most of the specimens were obtained from zoos, museums and biobanks in Europe. The specimens were either born locally in these EU institutions or in the wild and subsequently moved and/or collected by our collaborators under their respective licences. All sample donators and collaborators are listed in the Acknowledgements and in Tables [Link], [Link]. This research provides important tools not only to gain insights into illegal wildlife trade worldwide but also for preventing it. Furthermore, this work also addresses cryopreservation as a powerful conservation tool for the future. As such, this interdisciplinary project is helping to save species from extinction and conserve global biodiversity.

## Supporting information


**Table S1.** Sample donators and collaborators.

**Table S2.** NCBI genomes used for SNPSTR development.

**Table S3.** Primer data.

**Table S4.** SNPSTR data.

**Table S5.** List of media and cell growth conditions used for each taxonomic group.

**Table S6.** The FOGS database.

## Data Availability

*Genetic data*: Raw sequence reads and metadata are deposited in the SRA (BioProject PRJNA954578). *Sample metadata*: Metadata is also stored in the SRA (BioProject PRJNA954578).
