# Supplementary material for: FOGS: A SNPSTR Marker Database to Combat Wildlife Trafficking and a Cell Culture Bank for Ex‐Situ Conservation
Source: Mol Ecol Resour. 2025 Jan 10;25(4):e14062. doi: 10.1111/1755-0998.14062 (PMC11969641; doi:10.1111/1755-0998.14062)
Supplement: Supplementary file 1 — Table S1. Sample donators and collaborators. [file MEN-25-e14062-s003.pdf]

MOLECULAR ECOLOGY RESOURCES

Supplemental Information S1: Sample Donators and Collaborators

FOGS: a SNPSTR marker database to combat wildlife trafficking and a cell culture bank for *ex-situ* conservation

Annika Mozer, Camilla Bruno Di-Nizo, Albia Consul, Bruno Huettel, Richard Jäger, Ayodélé Akintayo, Christoph Erhardt, Lena Fenner, Dominik Fischer, Sophia Forat, France Gimnich, Peter Grobe, Sebastian Martin, Vikram Nathan, Ammar Saeed, Laura von der Mark, Christian Woehle, Klaus Olek, Bernhard Misof, Jonas J. Astrin

We thank all breeders and animal owners who provided samples.
Further we thank:

S. Dreyer, L. Grund, S. Lucki, L. Platner, J. Schwarzer, M. Stange
Leibniz Institute for the Analysis of Biodiversity Change Biobank
GBOL – German Barcode of Life Project
CaBOL – Caucasus Barcode of Life
CRARC – Centre de Recuperació d'Amfibis i Rèptils de Catalunya
Senckenberg Naturhistorische Sammlungen Dresden
Museum für Tierkunde
Soptom Station d'Observation et de Protection des Tortues et de leurs Milieux
Auffangstation für Reptilien, München
Greifvogelstation Wildtiergehege Hellenthal
CryoArks
Gesellschaft für Arterhaltende Vogelzucht e.V. (GAV)
Michael-Otto-Institut im NABU
Landesbund für Vogel- und Naturschutz in Bayern e.V. (LBV)
Totlok A.C. Conservación y Investigación de la Biodiversidad, Zoology Museum UNAM
Conabio Mexiko
Natural History Museum London
RZSS - The Royal Zoological Society of Scotland
Universität Bosnien und Herzegowina

Zemaljski Muzej Bosnie i Hercegovine
Universitats Belgradiensis
Universität Rostock
Italien League for Bird Protection
Universidad Nacional Autonoma Mexiko
Universität Lome
Ilia State University, Georgien
Institut für Fischerei Mecklenburg –Vorpommern
Herpetological Association in Bosnia and Hezegovina "ATRA"
Zoo Neuwied
Kölner Zoo
Wilhelma Stuttgart
Zoo Krefeld
Zoo Duisburg
Zoo Leipzig
Der grüne Zoo Wuppertal
Deutsche Gesellschaft für Herpetologie und Terrarienkunde
Mandai Nature, Singapur
South African National Biodiversity Institute - SANBI
National Science Museum Thailand
Bioplan Bühl
Bundesamt für Naturschutz
Untere Naturschutzbehörde Stadt Köln
Albera Turtle Breeding Center
