# Supplementary material for: FOGS: A SNPSTR Marker Database to Combat Wildlife Trafficking and a Cell Culture Bank for Ex‐Situ Conservation
Source: Mol Ecol Resour. 2025 Jan 10;25(4):e14062. doi: 10.1111/1755-0998.14062 (PMC11969641; doi:10.1111/1755-0998.14062)
Supplement: Supplementary file 2 — Table S2. NCBI genomes used for SNPSTR development. [file MEN-25-e14062-s005.pdf]

MOLECULAR ECOLOGY RESOURCES

Supplemental Information S2: NCBI Genomes used for SNPSTR development

FOGS: a SNPSTR marker database to combat wildlife trafficking and a cell culture bank for *ex-situ* conservation

Annika Mozer, Camilla Bruno Di-Nizo, Albia Consul, Bruno Huettel, Richard Jäger, Ayodélé Akintayo, Christoph Erhardt, Lena Fenner, Dominik Fischer, Sophia Forat, France Gimnich, Peter Grobe, Sebastian Martin, Vikram Nathan, Ammar Saeed, Laura von der Mark, Christian Woehle, Klaus Olek, Bernhard Misof, Jonas J. Astrin

Species	GenBank accession number:
Accipiter gentilis	GCA_012487025.1
Accipiter nisus	GCA_004320145.1
Acrocephalus scirpaceus	GCA_910950805.1
Alauda arvensis	GCA_902810485.1
Anguilla anguilla	GCA_013347855.1
Anguilla rostrata	GCA_001606085.1
Aquila chrysaetos	GCA_000766835.1
Bombycilla garrulus	GCA_013400315.1
Bubo bubo	GCA_010303855.1
Eretmochelys imbricata	GCA_015237465.2
Fringilla coelebs	GCA_015532645.1

MOLECULAR ECOLOGY RESOURCES

Grus monacha	GCA_012487855.1
Lacerta bilineata	GCA_900245895.1
Lacerta viridis	GCA_900245905.1
Lanius collurio	GCA_020086605.1
Loxodonta africana	GCA_030014295.1
Motacilla alba	GCA_013397355.1
Oenanthe oenanthe	GCA_013399995.1
Phataginus tricuspis	GCA_030020395.1
Phylloscopus trochilus	GCA_016584745.1
Podarcis muralis	GCA_004329235.1
Psittacus erithacus	GCA_009867235.2
Psittacula krameri	GCA_002870145.1
Recurvirostra avosetta	GCA_004023745.1
Sitta europaea	GCA_013400255.1
Sylvia atricapilla	GCA_009819655.1
Vipera berus	GCA_000800605.1
