# Supplementary material for: FOGS: A SNPSTR Marker Database to Combat Wildlife Trafficking and a Cell Culture Bank for Ex‐Situ Conservation
Source: Mol Ecol Resour. 2025 Jan 10;25(4):e14062. doi: 10.1111/1755-0998.14062 (PMC11969641; doi:10.1111/1755-0998.14062)
Supplement: Supplementary file 5 — Table S5. List of media and cell growth conditions used for each taxonomic group. [file MEN-25-e14062-s002.pdf]

MOLECULAR ECOLOGY RESOURCES

Supplemental Information S5: List of media and cell growth conditions used for each taxonomic group

FOGS: a SNPSTR marker database to combat wildlife trafficking and a cell culture bank for *ex-situ* conservation

Annika Mozer, Camilla Bruno Di-Nizo, Albia Consul, Bruno Huettel, Richard Jäger, Ayodélé Akintayo, Christoph Erhardt, Lena Fenner, Dominik Fischer, Sophia Forat, France Gimnich, Peter Grobe, Sebastian Martin, Vikram Nathan, Ammar Saeed, Laura von der Mark, Christian Woehle, Klaus Olek, Bernhard Misof, Jonas J. Astrin

List of media and cell growth conditions used for each taxonomic group

Taxonomic group	Media	Supplementation	Temperature
Amphibians	Leibovitz's L-15 Medium (Cytiva, Marlborough, USA)	10-20% FBS*	27°C
Birds	FBM™ Fibroblast Growth Basal Medium (Lonza, Cologne, Germany)	10-20% FBS*<br>5-10% AmnioMAX™II complete medium (Gibco) in slow growth cells	37°C / 5% CO_2
Fishes	Leibovitz's L-15 Medium (Cytiva)	10-20% FBS*	27°C
Mammals	Minimum Essential Medium (MEM, Sigma-Aldrich) or Dulbecco’s Modified Eagle Medium (DMEM, Sigma-Aldrich)	10-20% FBS*<br>5-10% AmnioMAX™II complete medium (Gibco) in slow growth cells	37°C / 5% CO_2
Reptiles	Leibovitz's L-15 Medium (Cytiva)	10-20% FBS*<br>5-10% AmnioMAX™II complete medium (Gibco) in slow growth cells	27°C

*FBS: Fetal bovine serum
