# Supplementary material for: FOGS: A SNPSTR Marker Database to Combat Wildlife Trafficking and a Cell Culture Bank for Ex‐Situ Conservation
Source: Mol Ecol Resour. 2025 Jan 10;25(4):e14062. doi: 10.1111/1755-0998.14062 (PMC11969641; doi:10.1111/1755-0998.14062)
Supplement: Supplementary file 6 — Table S6. The FOGS database. [file MEN-25-e14062-s004.pdf]

MOLECULAR ECOLOGY RESOURCES

Supplemental Information S6: The FOGS Database

FOGS: a SNPSTR marker database to combat wildlife trafficking and a cell culture bank for *ex-situ* conservation

Annika Mozer, Camilla Bruno Di-Nizo, Albia Consul, Bruno Huettel, Richard Jäger, Ayodélé Akintayo, Christoph Erhardt, Lena Fenner, Dominik Fischer, Sophia Forat, France Gimnich, Peter Grobe, Sebastian Martin, Vikram Nathan, Ammar Saeed, Laura von der Mark, Christian Woehle, Klaus Olek, Bernhard Misof, Jonas J. Astrin

Complex data structures are always a challenge for computer science. SNPSTR data with its hundreds of loci across multiple specimens is no exception. The data infrastructure set up in the FOGS project consists of three components: a central database for recording and managing all data, structured recording with automated database import and the publication of high-quality SNPSTR data on the FOGS portal with the option of downloading it.

The involvement of laboratories for SNPTSR generation, the LIB's own biobank and biodiversity informatics at the LIB required the development of a common understanding of the processes: what information is needed when and by whom, how the documentation obligation for Nagoya is fulfilled and what data is provided by the sequencing laboratory and in what form. In a second step, the specific data formats for the collections, the biobank and the laboratory were created. Data exchange between the laboratory and LIB takes place via data tables, which are exchanged via LIB's own Cloud storage. The laboratory's sequence data is also made available here for import into the central database. The Diversity Workbench Framewok[1] with the DiversityCollection database was selected as the database system, as collection management and results generation coincide in the FOGS project and Diversity Workbench is used at the LIB as a collection database and could be easily adapted to the requirements of the FOGS project.

1 https://www.diversityworkbench.net

MOLECULAR ECOLOGY
RESOURCES

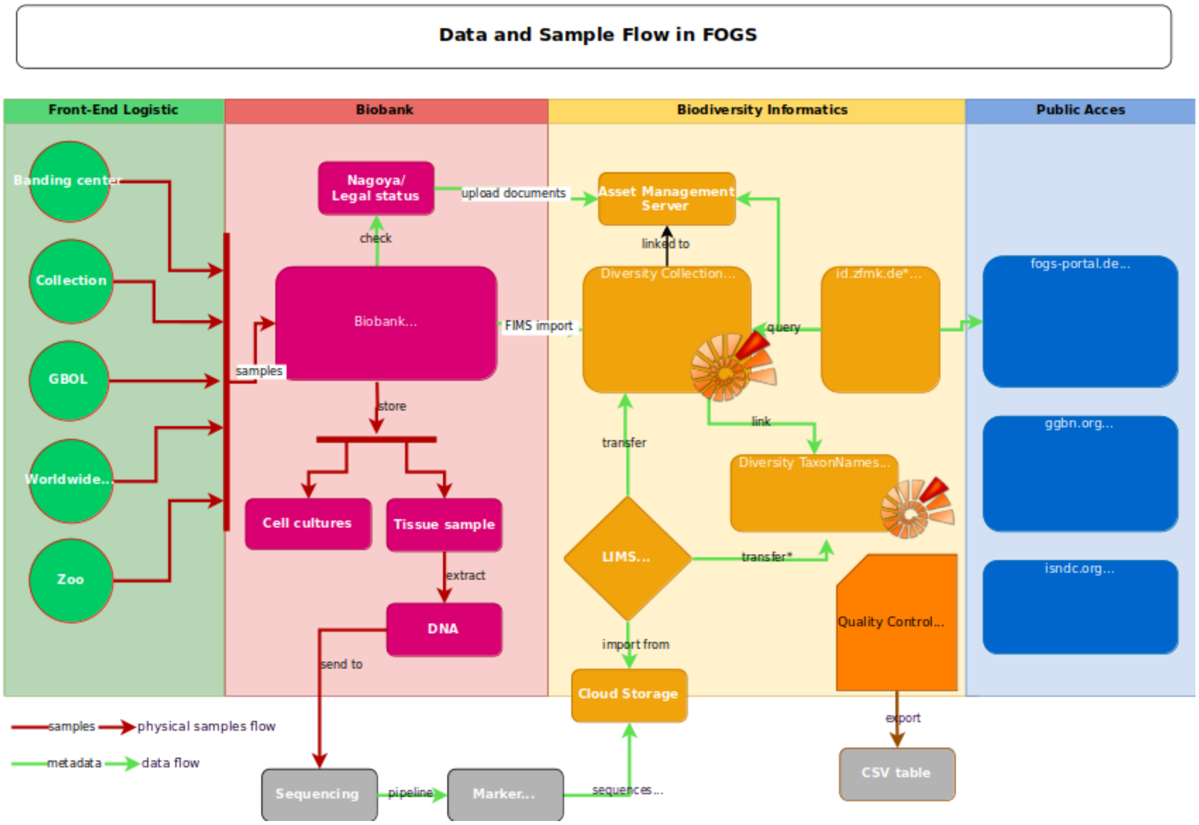

Fig.1: Dataprocessing in FOGS. Samples from the field, zoos, etc. are added to the LIB biobank and given a unique ID. The DNA extractions are sequenced and the FOGS analysis results are uploaded to the DiversityCollection database via cloud storage. Internal quality controls ensure the accuracy of the uploaded data. The final results are published on the FOGS data portal. The sequence data and the additional information on the species are uploaded to the Genbank[2] and the data is available in a broader context on the GGBN[3] portal.

The data flow is structured as follows: the analyzed SNPSTR data is stored as an Excel table in the LIB cloud (Fig. 2 top). From there, the tables are recorded by a script, validated and imported into DiversityCollection. The database contains one entry per specimen with a unique accession number. Collection and taxon information is appended to this and the corresponding data from the FOGs analysis (fig. 2 bottom). Each specimen of a species is analyzed using various informative gene loci relevant for the SNPSTR analysis. Each locus contains at least one (homozygous) and a maximum of two (heterozygous) entries with information about the mutations at certain sequence positions in comparison to a reference specimen. These are stored separately for each locus in the database for the specimen. In addition to the locus and the mutation, other stored information includes the repeat motifs, the reference allele and the inheritance type.

---

2 https://www.ncbi.nlm.nih.gov/

3 https://ggbn.org

MOLECULAR ECOLOGY RESOURCES

ZFMK-TIS	Species	Locus	Ref Amp size	Repeat motif	Ref allele	Haplotype										Inheritance
						67 A->G	80 C->T	85 G->C	94 A->C	107 G->A	133 Msat	266 C->A	267 T->C	282 C->T	296 A->G	
ZFMK-TIS-56653	Chersina angulata	CheAng13	350	[ATAG]6.3	[ATAG]11.3	-	-	-	-	-	6.3	-	-	-	-	he
ZFMK-TIS-56653	Chersina angulata	CheAng13	350	[ATAG]8.3	[ATAG]11.3	-	-	-	-	-	8.3	-	-	-	-	he
ZFMK-TIS-56656	Chersina angulata	CheAng13	350	[ATAG]11.3	[ATAG]11.3	-	-	C	-	-	11.3	-	-	-	-	he
ZFMK-TIS-56656	Chersina angulata	CheAng13	350	[ATAG]11.3	[ATAG]11.3	-	-	-	-	-	11.3	-	-	-	-	he
ZFMK-TIS-56658	Chersina angulata	CheAng13	350	[ATAG]16.3	[ATAG]11.3	-	-	C	-	-	16.3	-	-	-	-	ho
ZFMK-TIS-56658	Chersina angulata	CheAng13	350	[ATAG]16.3	[ATAG]11.3	-	-	C	-	-	16.3	-	-	-	-	ho
ZFMK-TIS-56661	Chersina angulata	CheAng13	350	[ATAG]9.3	[ATAG]11.3	-	-	-	-	-	9.3	-	-	-	G	he
ZFMK-TIS-56661	Chersina angulata	CheAng13	350	ATTG [ATAG]13.3	[ATAG]11.3	-	-	-	-	-	14.3b	-	-	T	G	he
ZFMK-TIS-56664	Chersina angulata	CheAng13	350	[ATAG]5.3	[ATAG]11.3	-	-	-	-	-	5.3	-	-	-	-	ho
ZFMK-TIS-56664	Chersina angulata	CheAng13	350	[ATAG]5.3	[ATAG]11.3	-	-	-	-	-	5.3	-	-	-	-	ho
ZFMK-TIS-59264	Chersina angulata	CheAng13	350	[ATAG]8.3	[ATAG]11.3	-	-	-	C	-	8.3	A	C	-	G	he
ZFMK-TIS-59264	Chersina angulata	CheAng13	350	[ATAG]11.3	[ATAG]11.3	-	-	-	-	A	11.3	A	C	-	G	he
ZFMK-TIS-59265	Chersina angulata	CheAng13	350	[ATAG]6.3	[ATAG]11.3	-	T	-	-	-	6.3	-	-	-	G	he
ZFMK-TIS-59265	Chersina angulata	CheAng13	350	[ATAG]8.3	[ATAG]11.3	G	-	-	-	-	8.3	-	-	-	-	he
ZFMK-TIS-59266	Chersina angulata	CheAng13	350	[ATAG]5.3	[ATAG]11.3	-	-	-	-	-	5.3	-	-	-	-	ho
ZFMK-TIS-59266	Chersina angulata	CheAng13	350	[ATAG]5.3	[ATAG]11.3	-	-	-	-	-	5.3	-	-	-	-	ho
ZFMK-TIS-59267	Chersina angulata	CheAng13	350	[ATAG]10.3	[ATAG]11.3	-	-	-	-	-	10.3	-	-	T	-	he
ZFMK-TIS-59267	Chersina angulata	CheAng13	350	[ATAG]13.3	[ATAG]11.3	G	-	-	-	-	13.3	-	-	T	-	he
ZFMK-TIS-59268	Chersina angulata	CheAng13	350	[ATAG]4.3	[ATAG]11.3	G	-	-	-	-	4.3	-	-	-	-	he
ZFMK-TIS-59268	Chersina angulata	CheAng13	350	[ATAG]4.3	[ATAG]11.3	-	-	-	-	-	4.3	-	-	-	-	he

CheAng1 CheAng2 CheAng3 CheAng4 CheAng5 CheAng7 CheAng9 CheAng12 CheAng13 CheAng14 CheAng15 CheAng16 CheAng17 CheAng19 CheAng20 CheAng23 CheAng24 CheAng26 CheAng27

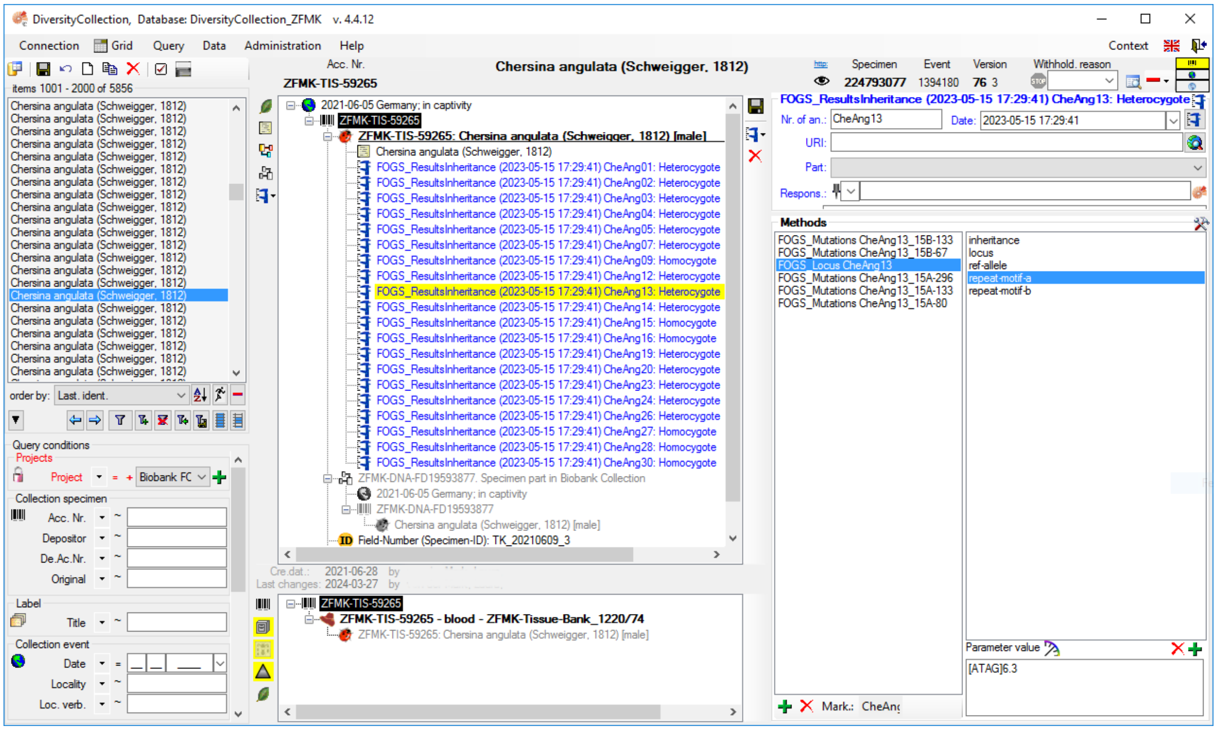

Fig. 2: Excel table for the structured recording of FOGS analysis results (top). The analysis results are stored in the DiversityCollection database (bottom). The same entry as in the Excel file is shown, with the value for repeat motif 1 of the heterozygous allele selected.
